# Green Synthesis of Silver Nanoparticles Using Aqueous *Citrus limon* Zest Extract: Characterization and Evaluation of Their Antioxidant and Antimicrobial Properties

**DOI:** 10.3390/nano12122013

**Published:** 2022-06-10

**Authors:** Yasmina Khane, Khedidja Benouis, Salim Albukhaty, Ghassan M. Sulaiman, Mosleh M. Abomughaid, Amer Al Ali, Djaber Aouf, Fares Fenniche, Sofiane Khane, Wahiba Chaibi, Abdallah Henni, Hadj Daoud Bouras, Nadir Dizge

**Affiliations:** 1 Université de Ghardaia, BP455, Ghardaia 47000, Algeria; 2 Laboratory of Applied Chemistry (LAC), DGRSDT, Ctr. Univ. Bouchaib Belhadj, Ain Temouchent 46000, Algeria; 3 Laboratory of Process Engineering, Materials and Environment, Department of Energy and Process Engineering, Faculty of Technology, University of Sidi Bel-Abbes, Sidi Bel Abbes 22000, Algeria; benouiskhadidja@yahoo.fr; 4 Department of Chemistry, College of Science, University of Misan, Maysan 62001, Iraq; 5 Department of Applied Sciences, University of Technology, Baghdad 10066, Iraq; 6 Department of Medical Laboratory Sciences, College of Applied Medical Sciences, University of Bisha, 255, Bisha 67714, Saudi Arabia; moslehali@ub.edu.sa (M.M.A.); ameralali@ub.edu.sa (A.A.A.); 7 Laboratory of Dynamic Interactions and Reactivity of Systems, University of Kasdi Merbah, Ouargla 30000, Algeria; djaberaouf@gmail.com (D.A.); fennichefares@yahoo.fr (F.F.); henni.abdallah@gmail.com (A.H.); 8 Department of Energy and Process Engineering, Faculty of Technology, University of Djillali Liabes, Sidi Bel Abbes 22000, Algeria; khanesofiane41@gmail.com; 9 Scientific and Technical Research Center in Chemistry and Physics Analysis, Bousmail RP 42415, Algeria; wahiba_chaibi@yahoo.fr; 10 Département de Physique, Ecole Normale Supérieure de Laghouat, RP Rue des Martyrs, Laghouat BP 4033, Algeria; hadjdaoud_bouras@yahoo.fr; 11 Department of Environmental Engineering, Mersin University, Mersin 33343, Turkey; nadirdizge@gmail.com

**Keywords:** biosynthesis, silver nanoparticles, *Citrus limon* zest extract, antibacterial properties, antioxidant activity

## Abstract

The current work concentrated on the green synthesis of silver nanoparticles (AgNPs) through the use of aqueous *Citrus* *limon* zest extract, optimizing the different experimental factors required for the formation and stability of AgNPs. The preparation of nanoparticles was confirmed by the observation of the color change of the mixture of silver nitrate, after the addition of the plant extract, from yellow to a reddish-brown colloidal suspension and was established by detecting the surface plasmon resonance band at 535.5 nm, utilizing UV-Visible analysis. The optimum conditions were found to be 1 mM of silver nitrate concentration, a 1:9 ratio extract of the mixture, and a 4 h incubation period. Fourier transform infrared spectroscopy spectrum indicated that the phytochemicals compounds present in *Citrus limon* zest extract had a fundamental effect on the production of AgNPs as a bio-reducing agent. The morphology, size, and elemental composition of AgNPs were investigated by zeta potential (ZP), dynamic light scattering (DLS), SEM, EDX, X-ray diffraction (XRD), and transmission electron microscopy (TEM) analysis, which showed crystalline spherical silver nanoparticles. In addition, the antimicrobial and antioxidant properties of this bioactive silver nanoparticle were also investigated. The AgNPs showed excellent antibacterial activity against one Gram-negative pathogens bacteria, *Escherichia coli*, and one Gram-positive bacteria, *Staphylococcus aureus*, as well as antifungal activity against *Candida albicans*. The obtained results indicate that the antioxidant activity of this nanoparticle is significant. This bioactive silver nanoparticle can be used in biomedical and pharmacological fields.

## 1. Introduction

The development of nanosciences in recent years has led to an explosion of thematic scientific research axes relating to the study of nanoparticles, along with the use of plants in the green biosynthesis of nanoparticles [1,2,3,4,5,6,7,8,9,10,11,12,13,14]. Compared to the chemical (photo-induced reduction, electrochemical deposition, microwave-assisted, etc.) and physical (laser ablation, high-energy irradiation, pyrolysis, etc.) techniques, biological pathways are fast, simple, economical, and, above all, effective and eco-friendly [15,16,17]. In fact, the compounds present in the extracts perform as potential reducing and capping agents [18,19,20]. They prevent the overgrowth of nanoparticles and minimize their aggregation in colloidal synthesis. These molecules can also influence and tailor the properties of the synthesized nanoparticles by improving their functionality in an optimal way for different applications [21,22]. In particular, plants with pharmacological properties have significant interest and potential for green AgNPs synthesis. Citrus is one of the medicinal plants with a wide number of pharmacological properties. It is one of the world’s most planted fruit crops, with an annual worldwide production of 124.3 million tons [23,24,25,26]. Citrus is the largest genus in the Rutaceae family, with around 70 species [27,28,29,30]. Because of their high levels of bioactive compounds, such as flavonoids, polyphenols, and vitamins [31,32], these fruits have significant antioxidant, anti-inflammatory, and anti-tumor activity [33,34], in addition to cardio and neuroprotective effects [35]. They represent an important source of minerals that reduce water retention and strengthen the bones and skeleton. After orange and mandarin, lemon or *Citrus Limon* (L.) is the third most significant *Citrus* species. In 2020/2021, global lemon production totaled approximately 8.4 million metric tons. Mexico was the world’s largest producer of lemons and limes; its yearly production is around 2.87 million metric tons [36]. Several studies have shown that *Citrus Limon* (L.) fruit cultivars could be employed to improve human health as antimicrobial and anti-oxidative [37,38], anti-inflammatory [39], and anti-tumor products [40]. It is very rich in bioactive substances, such as citric acid [41], phenolic compounds [42], flavonoids [43], carotenoids [44], and ascorbic acid [45]. Therefore, this study focused on the green fabrication of silver nanoparticles using the aqueous extract from the leaves of *Citrus limon*. The physicochemical characterization of the biosynthesized nanoparticles was carried out using UV-Vis, FTIR, XRD, DSL-zeta potential, SEM, and TEM technology. Furthermore, Gram-negative bacteria (*E. coli* ATCC 25922), Gram-positive bacteria (*S. aureus* ATCC 25923), and fungi (*Candida albicans*) were used to prove the antimicrobial properties of the AgNPs, and the antioxidant activity of this AgNPs and *Citrus limon* zest extract were investigated using the DPPH free radical scavenging method.

## 2. Materials and Methods

### 2.1. Experimental Parts

Silver nitrate (AgNO_3_, Cat. No. 209139), and DPPH (Cat. No.: D9132), were obtained from Sigma-Aldrich Co., St. Louis, MO, USA; bacterial and fungal nutrient agar and broth (Mueller-Hinton Cat. no. S3306) were obtained from Merck, Germany. All chemicals reagents were used as received, without any additional purification.

### 2.2. Selection of Plant

In January 2020, the fresh and adult *Citrus limon* (L.) were harvested randomly by hand when they were fully mature, from the same tree in a private-public farm in the area of Data Ben Dahoua, Ghardaïa, a Sahara Desert town located in northern-central Algeria. The selected fruits must be ripe, of yellow color, edible, and without any sign of infection. The fruit was washed three times with running water and then three times with deionized water, to remove any residual dust and black stains from the surface, and then air-dried at room temperature.

### 2.3. Preparation of the Citrus Limon Zest Aqueous Extract

The extract of *Citrus limon* zest was made by refluxing 15 g of rinsed and dried lemon zest in 150 mL of deionized water at 60 °C with shaking for 15 min. A clear pale yellow-colored solution was obtained. After allowing it to cool to room temperature, the extract solution was filtered with Whatman No.1 filter paper (Whatman, Fisher Scientific, Pittsburgh, PA, USA) to remove any suspended particles, and then the extract was divided into two parts: one part was stored at 4–8 °C to be utilized in the following step; the other part was dried in a vacuum oven for 48 h at 40 °C to obtain the powdered *lemon* zest extract for FTIR analysis.

### 2.4. Green Synthesis of Silver Nanoparticles

Green synthesis was carried out as previously explained, with minor modifications [46]. In a 250 mL flask, 10 mL of *Citrus lemon* zest extract was mixed with 90 mL of a concentration of 1 mM freshly prepared silver nitrate aqueous solution (AgNO_3_) and constantly stirred using a hot plate magnetic stirrer, with the rotation speed of 200 rpm at 60 °C under dark conditions. After 30 min, the mixture solution turned turbid and became reddish-brown, and the color of the colloidal suspension changed from yellow to brown, suggesting the formation of silver nanoparticles. In order to purify the AgNPs by removing the extract of *Citrus lemon* zest extract, the suspension was centrifuged three times at 15,000 rpm for 20 min to obtain a dark brown precipitate and washed twice with double sterilized water and once with methanol. Finally, the powder precipitate was dried to yield silver nanoparticles. To reach the optimum conditions for the production of AgNPs with *Citrus lemon* zest extract, we varied the various experimental factors such as the concentration of zest extract, the contact time, and the concentration of AgNO_3_, using UV-visible spectroscopy to examine the size and form of the controlled AgNPs.

### 2.5. Spectroscopy Characterization of Green AgNPs

The green AgNPs were analyzed using UV-Vis spectrophotometry (Thermo Fisher Scientific UviLine 9400C, Loughborough, UK) to investigate the surface plasmon resonance (SPR). FT-IR spectroscopy analysis was performed using an Agilent Cary 640 FTIR spectrometer, which was recorded to identify the functional groups and the surface chemistry of dried *Citrus limon* zest extract and synthesized silver nanoparticles in the range 400–4000 cm^−1^ at room temperature. The particle size and dynamic light scattering of AgNPs were calculated using a nano zeta sizer instrument (Malvern) at 25 °C with a 90° angle [47].

XRD measurement was performed using Philips (PW 1710) Diffractometer at (40 kV,40 mA), with Cu(Kα) radiation (λ = 1.5406 Å), in the range of the diffraction angle (2θ) from 10° to 90° to highlight the crystalline nature and purity of the AgNPs. The particle size of AgNPs was calculated using the Scherrer equation (D = Kλ/βcosθ) [48].

Where: D is the average crystallite size, K is the Scherrer constant, which ranges from 0.68 to 2.08, and 0.94 was used for cubic symmetry spherical crystallites, λ is the X-ray wavelength, CuKα = 1.5406 Å, β is the line broadening at FWHM (radians), and θ is the Bragg angle in degrees. Bragg’s equation (nλ = 2d sinθ, d is the light diffraction from the particles) was used for the calculation of the d-spacing value (light diffraction from particles) as recorded in Table 1. The morphology and the elemental composition of AgNPs were recorded using JSM-5910, JEOL scanning electron microscopy (SEM), combined with an energy dispersive X-ray spectrometer (EDX) and performed at 20 kV acceleration voltages, with a resolution of images ×30,000. AgNPs were also evaluated using the transmission electron microscope (TEM) (Zeiss EM 900 instrument model) to determine the morphological form, size, and shape of these silver nanoparticles.

### 2.6. Determination of Biological Activities of Silver Nanoparticles of Citrus Lemon Zest Extracts

#### 2.6.1. Antibacterial and Antifungal Activity

For comparison, the antimicrobial properties of AgNPs, silver nitrate, aqueous extract of *Citrus limon* zest, and standard antibiotics were studied using the agar well diffusion method [49] against human pathogenic bacteria, Gram-negative (*E. coli* ATCC 25922) and Gram-positive (*S. aureus* ATCC 25923), which were generously provided by the Laboratory of the Biology of Microbial Systems (Ecole Normale Supérieure of Kouba, Alger, Algeria), and one clinical fungal strain (*Candida albicans*) isolated from patient samples from the laboratory of Ghardaia Hospital (Algeria), and identified using conventional laboratory protocols. A total of 10^6^ CFU/mL (0.5 McFarland standards) of bacterial cultures in Mueller–Hinton broth were swabbed on the surface of an inoculated Mueller–Hinton agar plate using a sterile swab, and 10^6^ CFU/mL fungal cultures in Sabouraud dextrose broth were swabbed on the solidified SDA plates. After that, 6 mm wells were punched in the agar plate with a sterilized cork borer, and then each well was filled separately with 100 μL of AgNPs suspended in double sterilized water of 1 mg/mL, plant extract, and sterilized water as the negative control. For the positive control, gentamicin (100 μg) was used as a standard antibiotic against bacteria and nystatin (100 μg) against fungi for validating the method used. The standard antibiotic disc was deposed directly on the surface of the agar. A vernier caliper was used to estimate the width of an inhibitory zone formed by the sample after 24 h of incubation at 37 °C for bacteria and 30 °C for fungi, to express the antimicrobial activity. Each test was conducted in triplicate to confirm the results. 

#### 2.6.2. Antioxidant Activity

The AgNPs and *Citrus limon* zest extract were investigated for antioxidant activity using the DPPH free radical scavenging method, following the protocol of Lakhedari et al., with some modification, with the ascorbic acid serving as the positive control and the methanol as the negative control [50]. The DPPH radical-scavenging is a simple decoloration method. After the addition of the oxidized form of 1, 1-diphenyl-2-Picrylhydrazyl methanolic solutions (deep violet color) with the antioxidant compound, this causes the reduction of DPPH and the change of color from deep violet to yellow. A total of 2 mL of a standard solution of DPPH (0.1 mM) was added to 1 mL of synthesized AgNPs–*lemon* zest extract solution diluted with methanol to obtain various concentrations (100–500 μg/mL), along with the standard methanolic solutions of ascorbic acid. After that, the mixture was shaken and stored in the dark at room temperature. After 1 h of incubation, the results of radical scavenging activity were determined by measuring the absorbance (A) of each solution at 517 nm using a UV-visible spectrophotometer and estimating the percentage inhibition (I %) by using the equation as follows:

I% = [(A control sample − A test sample)/A control sample)] × 100

The antioxidant capacity was also evaluated by finding the IC_50_ value, which was the concentration of the test sample that could inhibit 50% of the DPPH radicals, and was computed in parallel from the linear plot of the ascorbic acid standard. 

## 3. Results and Discussion

In the present study, the aqueous solution of *Citrus lemon* zest extract was utilized as a bioactive green reducing agent for reducing silver ions to silver nanoparticles, due to phytochemical compounds present in the plant extracts, and the reaction process was monitored by spectroscopy analysis.

### 3.1. UV-Visible Spectral Analysis

**Figure 1:**
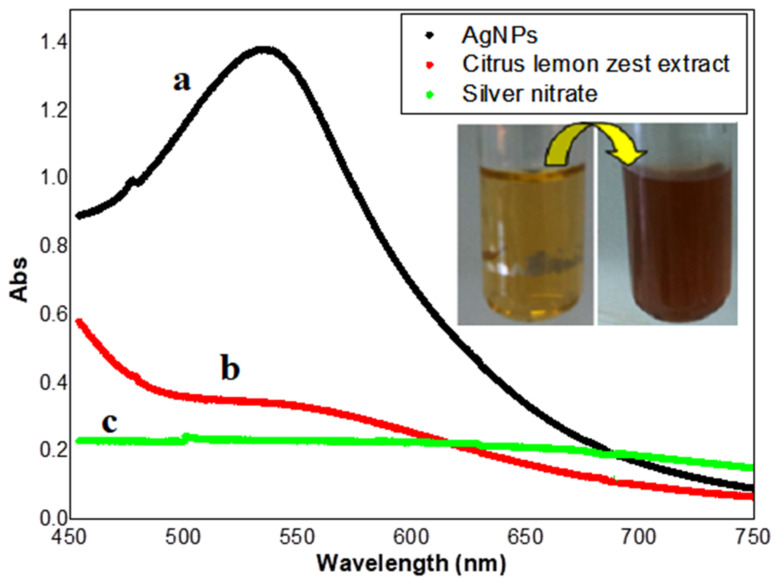
UV–Vis spectrophotometer analysis of biosynthesized AgNPs (**a**) *Citrus lemon* zest extract (**b**) *lemon* zest extract with silver nitrate (**c**).

The biosynthesis of AgNPs using *Citrus lemon* zest extract showed changes of color in the aqueous solution from yellow to reddish-brown, which was detected using a UV-Vis spectrophotometer, compared with the control sample (extract and silver nitrate solution), which exhibited no absorption band under the same conditions, as shown in Figure 1. The peak displayed at 535.5 nm represents surface plasmon resonance (SPR) due to the excitation of free electrons in metal during the synthesis of AgNP [51,52], which indicated that the optical characteristics of silver nitrate solution were changed because the silver ion was reducing to elemental silver, and finally, to silver nanoparticles, when exposed to the bioactive components of the plant extract [53].

### 3.2. Optimization of Different Experimental Factors in Biosynthesis of AgNPs

**Figure 2:**
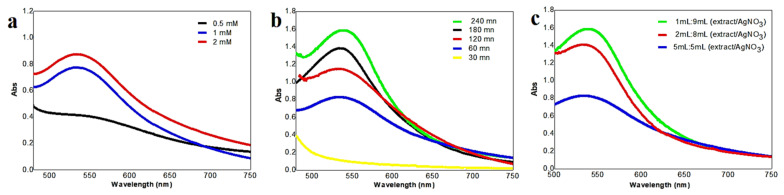
UV-Visible spectrum of the optimizing factor nanoparticles: (**a**) concentration of silver nitrate; (**b**) the contact time; (**c**) concentration of *Citrus lemon* zest extract.

Several different experimental parameters, including the incubation time of the reaction, the concentration of AgNPs, and the extract volume play, an essential role in the synthesis and stabilization of AgNPs with different characteristic properties. This effect was controlled by comparing the surface plasmon resonance (SPR) band at 535.5 after analyzing the AgNPs synthesized at different parameters with a UV-Vis spectrophotometer, as shown in Figure 2.

The effect of incubation periods: Figure 2a clearly demonstrates that the absorbance of the mixture increased as the contact time increased from 30 min to 240 min, due to the evolution of the reduction in silver ions and the increase in the number of silver nanoparticles [54]. The UV-Vis spectra exhibited strong SPR absorbance after 240 min due to the stabilization of nanoparticles because of the stabilization agent present in the plant extract [55]. Then, the rate of reaction of the lemon zest mediated silver nanoparticles is 4 h, which is faster compared with the biosynthesis of AgNPs using *Citrus tangerina*, *Citrus sinensis*, and *Citrus limon* peel extracts [56]. The effect of silver nitrate concentration: the influence of various AgNO_3_ concentrations in the production of AgNPs was investigated by mixing each concentration of AgNO_3_ solution (2, 1, 0.5 mM) with 9 mL of *Citrus limon* zest extract, and other parameters remained the same as those used in the previous experiments. As shown in Figure 2b, the surface plasmon frequency gradually increased as the concentration of AgNO_3_ increased from 0.5 mM to 2 mM, which means that the higher concentration of AgNO_3_ solution increased the silver nanoparticle size [57]. It is clear that the three concentrations permit the synthesis of AgNPs, but when using the 2 nM concentration, the particles show aggregation and precipitation in the bottom of the flask. The optimum concentration was then found to be 1 nM, which gave the highest yield of nanoparticles compared to the other concentrations and showed less toxicity in the same experiment. The effect of *Citrus limon* zest extract quantity: the green synthesis of AgNPs was carried out by varying the extract ration (1 mL, 2 mL, and 5 mL) in the reaction mixture using the same biosynthesis process and fixing the optimum contact time and the quantity of *lemon* zest extract, which were selected in the previous experiments. During the reaction of the preparation of silver nanoparticle with a varying quantity of *C. limon* zest extract from 1 mL to 5 mL, the yellow color of the mixture changed to dark brown. As shown in Figure 2c, the UV-Vis spectra of AgNPs synthesis with different quantities recorded a decrease in the intensity of the SPR band with the increase in the quantity of the extract added to the AgNO_3_, and the maximum plasmon absorption was obtained for the mixture of 1:9, (extract:AgNO_3_). Thus, the formation of the nanoparticle is considerably favored at the lower quantity of lemon zest extract, due to the presence of sufficient biomolecules necessary for the reduction and stabilization of 10^−3^ M of AgNO_3_ aqueous solution. The same results were achieved for the biosynthesis of AgNPs using *P. guajava* leaf extract [58].

### 3.3. FTIR Analysis

**Figure 3:**
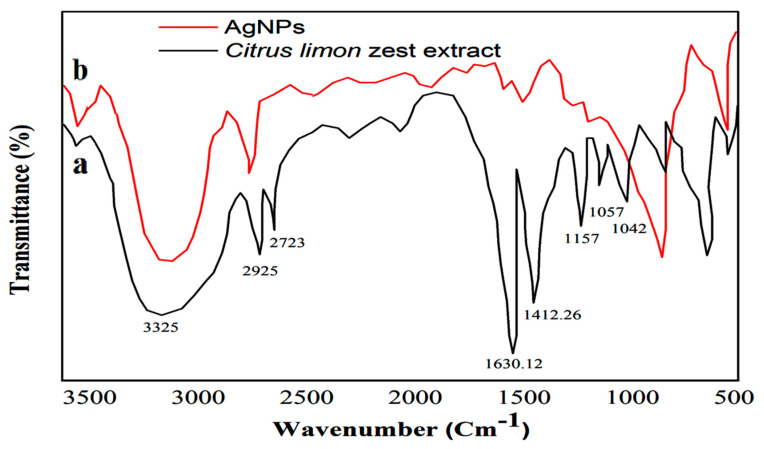
FTIR spectrum of (**a**) *Citrus lemon* zest extract and (**b**) silver nanoparticle.

As shown in Figure 3, the FTIR spectrum of the dried *Citrus* *lemon* zest extract and the synthesis AgNPs provide information about the biofunctional groups involved in the bioreduction of silver. Generally, the FTIR spectra of the plant extract (Figure 3a) detected the presence of absorption peaks located at 1042.18, 1157, and 2723 cm^−1^ corresponded to –C–O groups of the polyols, such as the flavones and polysaccharides, N–O stretching, and the C-H stretching of the methylene groups, respectively. Weaker peaks at 1412.26 and 1630.121 cm^−1^ were related to the N-H bond of primary amines and the carbonyl stretch –C=O, respectively. The peak at 1634 cm^−1^ and the intense band at 3325 cm^−1^ were attributed to the amide functional groups of diverse aromatics and carbonyl groups of proteins. The C–N stretching of amines appeared at 1057 cm^−1^, whereas the band around 3431 cm^−1^ indicates the presence of hydroxyl groups (OH) in the compound, and a peak at 2925 cm^−1^ reflects the presence of the aliphatic hydrocarbons chains, C–H group stretching of alcohol, carboxylic acid, and phenolic compounds [59]. After the addition of AgNO_3_ aqueous solution with bio extract, the FTIR spectrum revealed a significant change in absorption peaks at 1021, 1443, 1634, and 3428 cm^−1^, which confirms that the functional groups interacted with the surface of the AgNPs. The biomolecule compounds, such as polyphenol, and their derivatives, including tannic acid, flavonoids, phytosterols, and phenolic compounds, served as reducing agents of silver ion to silver nanoparticles because of their oxidation-reduction potential [60]. A recent study suggests that plant metabolites, such as flavonoids, phenols, and aromatic compounds soluble in the aqueous extract, exhibit remarkable performance in the bioreduction of Ag ions and the stabilization of silver nanoparticles [61]. The bioreduction process of a silver ion with phytochemical compounds present in plant extracts of AgNPs is still unknown. 

### 3.4. XRD Analysis

**Figure 4:**
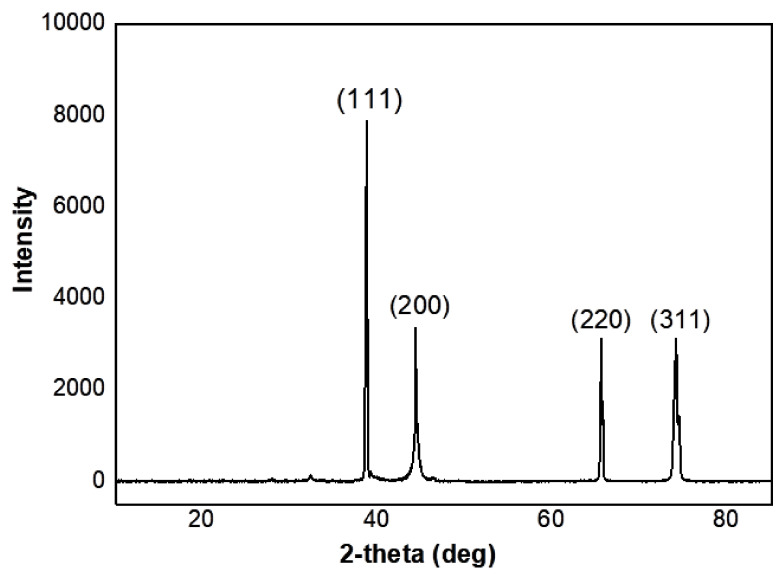
XRD pattern of AgNps synthesized using *Citrus limon* zest extract.

The XRD method was utilized to highlight the crystal structure of synthesized AgNPs using an aqueous extract of *Citrus lemon* zest, and confirm the UV-Vis analysis (Figure 4). Diffraction peaks at 2θ of 38.18, 44.34, 64.53, and 77.46 can be ascribed to the crystallographic planes (1 1 1), (2 0 0), (2 2 0) and (3 1 1), respectively. The resulting diffraction pattern confirms that the AgNPs have a face-centered cubic structure, and also confirms the crystalline nature of AgNPs [62]. The calculated particles size of AgNPs using the Scherrer equation was equal to approximately 15.98 nm. Similar results have been established by Sadeghi and Gholamhoseinpoor [63], Moira et al. [56], and Devanesan and AlSalhi [64] for the biosynthesis of AgNPs using *Ziziphora tenuior*, citrus fruits (*Citrus tangerine*, *Citrus sinensis*, and *Citrus limon*), as well as *Abelmoschus esculentus* plant extracts, respectively. Similarly, Sujitha et al. [65] previously reported on the formation of gold nanoparticles using aqueous extracts of citrus fruits (*citrus limon*, *citrus reticulata*, and *citrus sinensis*).

### 3.5. Zeta Potential and DLS Characterization

Particle size distribution and zeta potential values of the silver nanoparticle solution were evaluated using a nano zeta sizer, and dynamic light scattering. 

**Figure 5:**
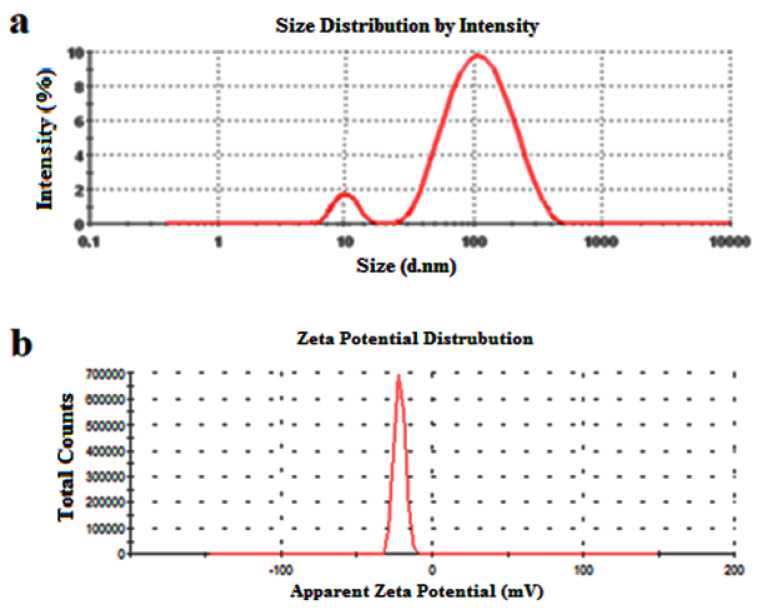
(**a**) DLS and (**b**) zeta potential analysis of silver nanoparticles produced using *Citrus Limon* zest leaf extract in water at 25 °C using Zetasizer^®^ software.

Dynamic light scattering is used to determine the thickness of the capping or stabilizing compound enveloping metallic particles, as well as the average size distribution of the AgNPs in the solution, which was found to be 82.51 nm, and the polydispersity index (PDI), which was found to be 0.248 (Figure 5a and Table 2). We can observe that the AgNPs have a PDI value lower than 0.7, which indicates the good quality of these synthesized silver nanoparticles using *Citrus lemon* extract and the relatively well-defined dimensions, with high monodispersity (PDI of 0.248). The zeta potential value was calculated to determine the surface charge of the synthesized AgNPs and to quantify the magnitude of charge, which proved the stability of nanoparticles in dispersion by the development of certain charge groups on their surface [66]. The result (Figure 5b and Table 2) showed that the AgNPs produced by *Citrus limon* zest extract exhibit a negative charge of −21.5 mV, possibly due to the adsorption of free nitrate ions present in the mixture, which provides the repulsive force as electrostatic stabilization [67]. Salvioni et al. [68] published a study exhibiting a mainly negative charge of AgNPs synthesized with citric and tannic acid.

### 3.6. SEM-EDX and TEM Analysis

**Figure 6:**
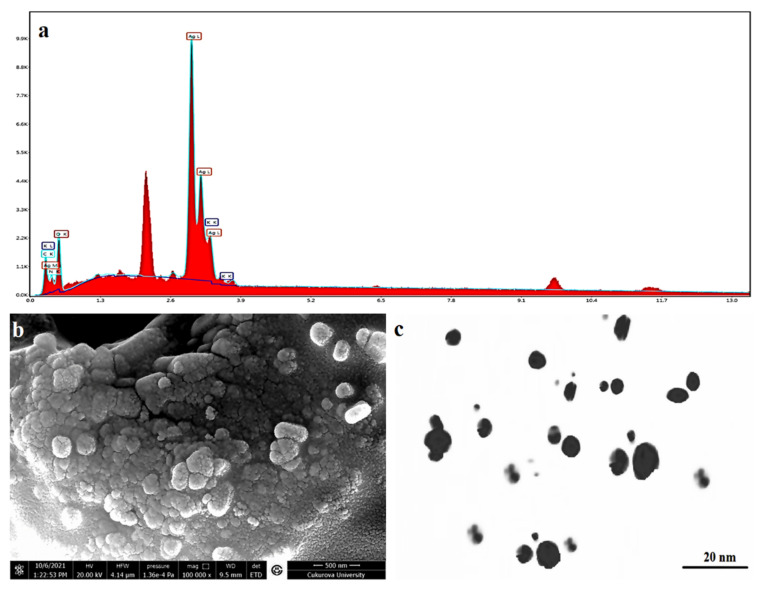
(**a**) EDX, (**b**) SEM micrographs, and (**c**) TEM imaging of AgNPs synthesized using *Citrus limon* zest extract.

The EDX spectrum is mainly used for identifying the elemental composition, and purity of the biogenic synthesized AgNPs, as shown in Figure 6a and Table 3. A strong signal for Ag, with high atomic percent values, was noted at 2 keV, which confirms the formation of AgNPs synthesized with an aqueous extract of *Citrus* *limon* zest. Additionally, a few weak signals of C, O, N, and K were also obtained, attributed to the existence of plant bioactive molecules that are linked to the surface of the AgNPs. As shown in Figure 6b,c, the morphological pattern of the biosynthesized AgNPs of *Citrus limon* zest extract was mainly characterized using SEM micrographs and TEM, which recorded that the nanoparticle had a spherical particle, relatively face-centered cubic shape, with different sizes between 7–28 nm, adjusted using XRD analysis and DLS results. The same result was reported in some previous studies [69].

### 3.7. Biological Activity

#### 3.7.1. Antioxidant Activity Using DPPH Method

The antioxidant activity of the AgNPs and the aqueous extract of *citrus limon* zest were evaluated utilizing DPPH free radical scavenging and using ascorbic acid as the standard to prepare the range of calibration. 

**Figure 7:**
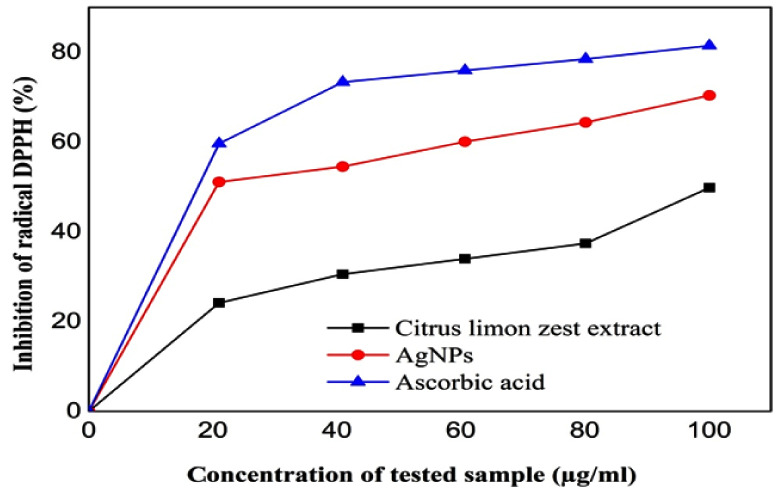
Percentage of inhibition of DPPH free radicals with different concentrations of *Citrus limon* zest extract, AgNPs, and ascorbic acid.

As shown in the Table 4 and Figure 7, there is a difference in antioxidant potential between the extracts and the silver nanoparticles; both samples reacted directly and reduced the wide range of free radicals of DPPH [70], and the scavenging rate increased as the concentration of the tested sample increased (45.39 ± 2.16%, 53.69 ± 3.84%, and 68.56 ± 2.41% at 25, 50, and 100 µg/mL) with lemon zest extract and (22.29 ± 0.79%, 30.59 ± 1.95%, and 54.11 ± 2.03% at 25, 50, and 100 µg/mL) with green AgNPs. In addition, the biosynthesis of silver nanoparticles using *Citrus limon* zest extract displayed the best antioxidant activity, with IC_50_ of about 42.56 ± 0.02 μg/mL, compared with 84 ± 0.079μg/mL for the plant extract. However, the silver nanoparticle synthesized with *Citrus limon* zest extract showed a stronger antioxidant capacity than the extract, and this difference may be because of the chemical structure of each sample tested [71].

Previous studies indicate that the *Citrus limon* zest extract exhibits significant antioxidant activity due to the redox properties of their important natural antioxidants, such as phenolic acids and flavonoids [72,73]. On the other hand, the antioxidant capacity of AgNPs might be related to the presence of phenolic compounds and flavonoids that form a coating over the silver nanoparticle, and it may also be higher due to the synergistic effect of the nanosized silver nanoparticles after the physicochemical interaction of Ag ions with the functional groups of the lemon zest extract, which formed a spherical shape with a larger surface area [74].

Since both silver nanoparticles and *Citrus limon* zest extract showed a significantly different performance compared with ascorbic acid as a standard solution, we can say that this work highlights the therapeutic value of AgNO_3_ synthesized by *Citrus limon* zest extract as a source for antioxidant drug development for medical care.

#### 3.7.2. Antimicrobial Activity

The antibacterial and antifungal activity of the sample was evaluated in vitro against four microbial strains, including one Gram-negative bacteria (*Escherichia coli* ATCC 25922); one Gram-positive bacteria (*Staphylococcus aureus* ATCC 25923), and one yeast (*Candida albicans*) using the well agar diffusion method, and the different diameters of inhibition zones were recorded in Table 5. According to Ponce et al. [75], the bacterial sensitivity toward the test sample was classified into four levels of activity: (resistance D < 8 mm, sensitive 9 mm ≤ D ≤ 14 mm, very sensitive 15 mm ≤ D ≤ 19 mm, extremely sensitive D >20 mm).

(a) Antibacterial Activity:

Regarding this study, the results in Table 5 revealed that the *Citrus limon* aqueous extract has no antibacterial activity, and no inhibition zone was observed on the discs after the 24 h of incubation with all the bacterial tested, and there was no inhibition zone around the well filled with silver nitrate, due to the low concentration of silver nitrate (1 mM) [76,77]. 

Recent research studies have proved that the polyphenols, including the phenolic acids and flavonoids, are the major plant bioactive compounds in *Citrus limon* zest extract [78,79] that act as a natural antibiotic and provide significant bactericidal activity against human pathogens, including fungi, yeasts, and bacteria [80] Furthermore, the silver nanoparticle coated by these biomolecules exist in the lemon extract, giving it biocidal activity [81]. The activity of the NPs change, depending on the stabilizer used and the functional groups that could be attached to its surface [82]. 

On other hand, the prepared AgNPs using aqueous extract of lemon zest have shown a considerable bactericidal ability toward both Gram-negative and Gram-positive bacteria, with inhibition zones ranging between 14 mm and 20 mm; the greatest antibacterial capacity was achieved against *E. coli*, with maximum inhibitory zone diameters of 20 mm compared with *S. aureus*, with 14 mm. According to this result, Gram-negative strains were more susceptible than Gram-positive strains [83]. This is probably because of the thin peptidoglycan layer in Gram-negative bacteria, as well as an extra outer membrane made of lipopolysaccharide, implying the presence of a periplasmic membrane layer. It is possible that this structure could make the entry of NPs and released ions inside the cell easier. On the other hand, there is a thick layer of peptidoglycan in Gram-positive bacteria’s cell walls that includes covalently linked teichoic and teichuronic acids, which might possibly act as a protective covering against the inhibitory effects of AgNPs and Ag^+^ [84]. Furthermore, Gram-negative bacteria are more sensitive to cell wall breakdown because of their interactions with NPs. This bacterium is covered in negative-charged lipopolysaccharide molecules, which have a stronger affinity for positive nanoparticles and released ions, leading to ion buildup and absorption, which causes intracellular damage [85].

Furthermore, the AgNPs presented no significantly difference compared with the standard antibiotic (gentamicin), which exhibited inhibition zones of 25 mm and 22 mm against *S. aureus* and *E. coli*, respectively. This may be because the silver nanoparticles can often penetrate cells, increasing their intracellular persistence as a result of their high permeability [86]. Even with this encouraging result, there is a dispute over the inhibitory mechanisms.

(b) Antifungal Activity:

The yeast *Candida albicans* is one of the normal human microbiomes that can exist in human tissue and inside organs such as the mouth, throat, gut, and vagina without causing any health problems [87]. However, under certain conditions, *C. albicans* can become a pathogenic fungus if it grows out of control or penetrates deeply into the body. This yeast is the most prevalent human fungal infection [88], which can cause effects ranging from serious superficial mucosal infections to life-threatening systemic infections [85]. Besides, this fungus can also foster the growth and colonization of other bacteria, which can lead to more severe infectious illnesses and the development of antibiotic drug resistance. For this purpose, the *Citrus limon* extract and the AgNPs were also evaluated for their antifungal ability against the *Candida albicans* fungal strain. The test indicated the presence of *C. albicans* growth around the sterile disc impregnated with *Citrus limon* zest extract, which indicates that this extract does not contain any antifungal agents. However, the AgNPs were found to have a strong fungicidal activity, with inhibition zone diameters of 20 mm, but a lower effect compared with nystatin as a positive control, which indicated a zone of inhibition of 32 mm.

(c) Antibacterial mechanisms of AgNPs

**Figure 8:**
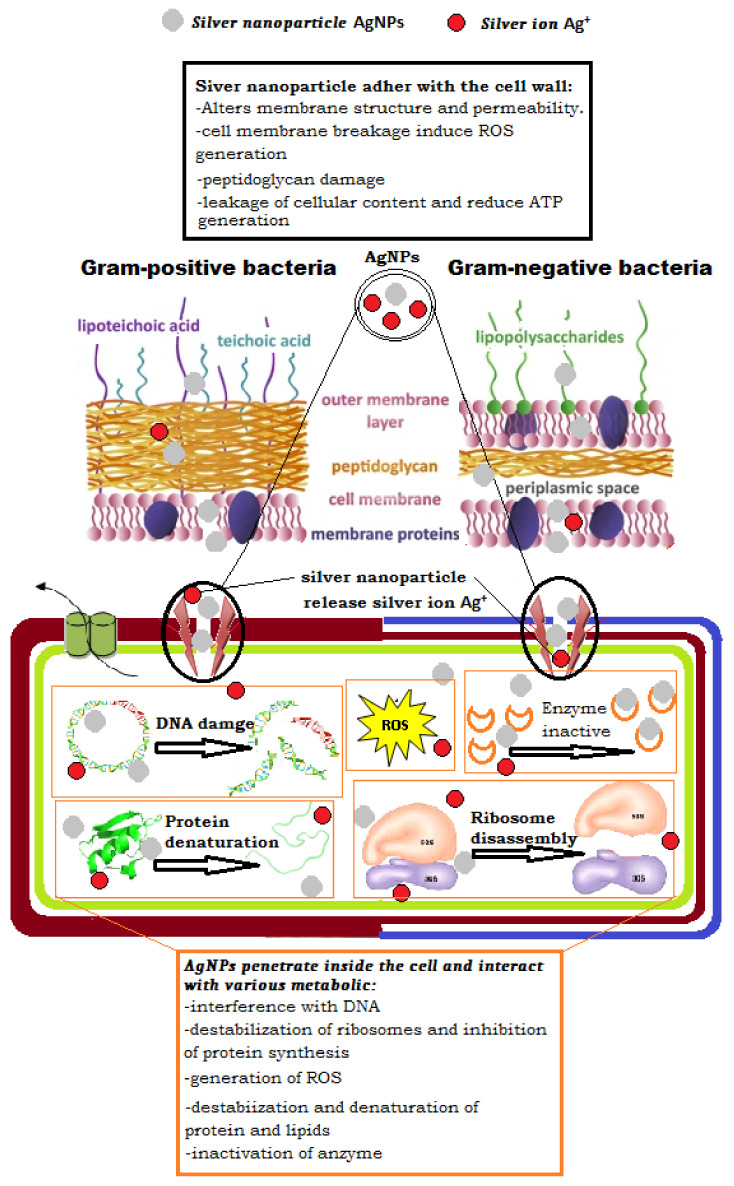
Various proposals for the antibacterial mechanism of silver nanoparticles.

Many previous researchers have used different microscopic methods to investigate the effects of treatment with AgNP on the cell wall’s surface structure and mechanical properties and the subsequent breakdown of the bacterial cell membrane [89]. As illustrated in Figure 8. They have suggested a different mechanism of the silver nanoparticle inhibitory effect, depending on the adhesion of the spherical AgNPs in the cell membrane of microorganisms, as well as its penetration and release into the bacterial cells [90,91].

Many authors have suggested various antibacterial mechanisms of silver nanoparticles; according to Yassin, Mohamed Taha, et al., they can continuously release Ag ions, which could be a microbe-killing mechanism. Ag ions can attach to the cell wall and cytoplasmic membrane, due to electrostatic interaction and their affinity for sulfur proteins [92]. The attached ions can increase the permeability of the cytoplasmic membrane, causing the bacterial envelope to be disrupted, according to a study by Bilal Ahmed et al. [93]. When free Ag ions are introduced into cells, respiratory enzymes are inhibited, resulting in reactive oxygen species that can be a principal agent in the provocation of cell membrane disruption and deoxyribonucleic acid (DNA) modification; moreover, silver ions can inhibit the synthesis of proteins by denaturing ribosomes in the cytoplasm, and the dissolution condition of AgNPs in exposure media significantly affects their antimicrobial effect and mechanism. The dissolution efficiency depends on synthetic and processing parameters, such as intrinsic AgNPs properties and surrounding media [94]. The primary mechanism suggested that the AgNPs are exposed to bacterial cells, which is often accompanied by penetration into the cell, which can cause membrane damage [95,96]. According to this hypothesis, the silver nanoparticles are concentrated around the bacterial cell membrane, with dissolving kinetics dependent on the size and form of the nanoparticle [97]. Most significantly, a positive charge in the NPs has been found to increase toxicity because the positively charged AgNPs are electrostatically attracted to the negative charge of the bacterial cell wall, enhancing their effectiveness [98]. Because of the positive charge of AgNPs, the function of the bacteria’s electron transport chain is changed [99]. 

According to some theories, NP adsorption damages the cell by first interacting with it, forming a barrier between the cell wall and the cytoplasm and abnormal pit formations on the cell wall, which causes the depolarization and change of its negative charge to make it more permeable, allowing ions to easily enter the cell, disrupting transport control [100,101]. Subsequently, the extracellular ROS is produced, which inhibits ATP generation and DNA replication, disrupting the cell’s built-in antioxidant defense and causing an increase in cell wall damage [102,103] or cell death [104].

On the other hand, many studies have reported that the antibacterial activity of NPs is frequently caused by the release of ions, such the silver ions, that are often responsible for antimicrobial toxicity, but other factors and mechanisms may also be involved [105]. Due to their electrochemical potential, NPs are dissolved in solutions and release metal ions that interact with a bacterial cell and become uniformly dispersed in the bacterial cell, with no specific localization [106,107]. Furthermore, Ag+ has been proposed to enter the cell via cation-selective porins, providing another potential pathway for Ag+ to enter the cell to disrupt the process of cellular respiration and produce toxicity [108].

Additionally, some research has demonstrated that the NPs surface morphology has a significant impact on their activity; the small size and high surface of NPs aid in their penetration of the cells [109]. NPs with a small diameter and the silver ion with high concentration appear to be able to enter the bacterial intracellular area [110]. Many studies have demonstrated that the cells exposed to AgNPs can generate more intracellular ROS than cells exposed only to silver ions, inducing oxidative stress [111]. However, Ag^+^ blocks the site and increases ROS, which can cause cellular DNA damage without obvious membrane disruption, suggesting a complicated toxicity method for inhibiting bacterial growth or cell death [112]. 

Other investigations discovered DNA damage, in which it lost its replication ability [113,114]. This damage comprised nuclear fragmentation [115] and physical attachment of silver nanoparticles with DNA, which was likely caused by silver ion’s high affinity for phosphates, found in high abundance in DNA molecules [116]. Another study reported that the majority of proteins were found to be involved in central metabolism, as well as genetic transcription, and other cellular processes such as membrane construction and biofilm production.

The ribosomal subunit proteins, as well as other cellular proteins and enzymes involved in ATP synthesis, were inactive when a bacterial cell was treated with AgNPs and Ag^+^. As a result, the ribosome lost its function and became completely denatured [117].

## 4. Conclusions

*Citrus limon* zest extract is very rich in bioactive molecules, including phenolic acids, citric acid, ascorbic acid, flavonoids, and minerals. For this purpose, we used a green, nontoxic and simple technique for the biosynthesis of silver nanoparticles by *Citrus limon* zest extract and optimized the different experimental factors, including the concentration of the extract, the contact time, and the metal ion concentration required for the formation and stability of AgNPs. The phytochemicals present in lemon zest aqueous solution, including flavonoids and phenolic compounds, may possibly be responsible for the reduction of silver nitrate to silver ions, and subsequently, aggregate to silver nanoparticles at the nanoscale range, and have a stronger ability to efficiently stabilize the synthesis of AgNPs. These biomolecules have been used as a natural reducing and stabilizing agent, as they envelope the core of the AgNPs. According to the results of this study, the silver nanoparticles synthesized using *Citrus limon* zest extract showed a very interesting ability to reduce pathogenic bacteria and fungi, which highlights the therapeutic value of these particles as antimicrobial and antioxidant agents against antibiotic drug-resistant strains and for use in medical applications.

## Figures and Tables

**Table 1:** XRD analysis data and further shape description using the Bragg equation (2dsinθ = nλ) and the Scherrer equation D = Kλ/βcosθ.

Peak Number		Scherrer Equation			Bragg Equation
	Peak Position 2θ (°)	FWHM β (°)	D (nm)	Average D (nm)	d Space
1	38.17753	0.45325	19.37	15.98	2.355
2	44.34242	0.67363	13.30		2.041
3	64.53204	0.60943	16.10		1.443
4	77.46486	0.7025	15.14		1.231

**Table 2:** Surface zeta potential values and particle size distribution of AgNPs.

T	Conductivity	Zeta Potential (ZP)	Zeta Deviation	z-Average Size	Polydispersity Index (PDI)
°C	mS/cm	mV	mV	(d.nm)	
25	0.158	−21.5	6.20	82.51	0.254

**Table 3:** Elemental composition of AgNPs synthesized with aqueous *Citrus* *limon* zest extract.

Element	Weight %	Atomic %	Net Int.
**C (K)**	4.14	10.43	110.91
**N (K)**	5.18	11.2	45.34
**O (K)**	31.65	59.93	331.45
**Ag (L)**	55.28	15.53	2273.71
**K (K)**	3.75	2.91	268.93

**Table 4:** IC_50_ values of *Citrus limon* zest extract, AgNPs, and ascorbic acid.

	*Citrus limon* Zest Extract	AgNPs	Ascorbic Acid
**IC_50_ (µg/mL)**	84 ± 0.079	42.56 ± 0.02	22.6 ± 0.06

**Table 5:** Antimicrobial activity of AgNPs synthesized with *Citrus Limon* fresh zest extract against human bacterial pathogens.

		Bacteria Strains		Fungi
		Gram-Positive	Gram-Negative	
		*S. aureus*	*E. coli*	*C. albicans*
**AgNO_3_**		**8**	**11**	**12**
		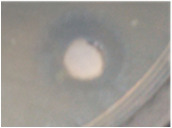	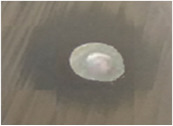	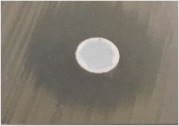
***Citrus limon* extract**		**-**	**-**	**8**
		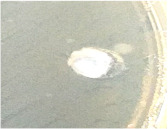	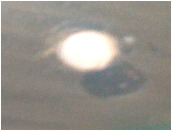	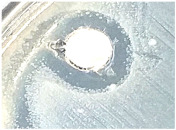
**Ag NPs**		**14**	**20**	**24**
		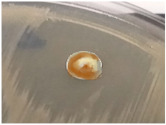	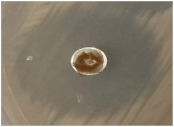	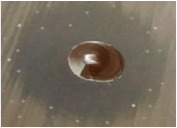
**Negative control**	**Distilled Water**	**0**	**0**	**0**
**Positive control**	**Gentamicin**	**22**	**25**	**-**
	**Nystatin**	**-**	**-**	**32**

*No zone of inhibition; mean values ± standard deviation (mm).*

## Data Availability

Not applicable.
